# Quantitative Detection of Weak D Antigen Variants in Blood Typing using SPR

**DOI:** 10.1038/s41598-017-01817-x

**Published:** 2017-05-09

**Authors:** Whui Lyn Then, Marie-Isabel Aguilar, Gil Garnier

**Affiliations:** 1 Bioresource Research Institute of Australia (BioPRIA), Australian Pulp and Paper Institute (APPI), Department of Chemical Engineering, Faculty of Engineering, Monash University, Clayton, VIC 3800 Australia; 2 Monash biomedicine Discovery Institute and Department of Biochemistry and Molecular Biology, Faculty of Medicine, Nursing and Heath Sciences, Monash University, Clayton, VIC 3800 Australia

## Abstract

Modern techniques for quantifying blood group antibody-antigen interactions are very limited, especially for weaker interactions which result from low antigen expression and/or partial expression of the antigen structure. Surface plasmon resonance (SPR) detection is often used to monitor and quantify bio-interactions. Previously, a regenerable, multi-fucntional platform for quantitative RBC phenotyping of normal antigen expression using SPR detection was reported. However, detection of weaker variants were not explored. Here, this sensitivity study used anti-human IgG antibodies immobilized to a gold sensor surface to two clinically important types of weaker D variants using SPR; weak D and partial D. Positive pre-sensitised cells bind to the anti-human IgG monolayer, and the response unit (RU) is reported (>100 RU). Unbound negative cells are directly eluted (<100 RU). Weak D cells were detected between a range of 180–580 RU, due to a lower expression of antigens. Partial D cells, category D VI, were also positively identified (352–1147 RU), similar to that of normal D antigens. The detection of two classes of weaker D variants was achieved for the first time using this fully regenerable SPR platform, opening up a new avenue to replace the current subjective and arbitrary methods for quantifying blood group antibody-antigen interactions.

## Introduction

Mismatching incompatible blood types can lead to a haemolytic transfusion reaction, the severity of which can range from mild to fatal^1^. Therefore, accurate and reliable blood typing is essential prior to any blood transfusion. Current blood typing methods available are well established. The column agglutination test (CAT), is the most common qualitative technique for blood group antigen identification. However, methods for quantifying blood group antibody-antigen interactions are currently very limited. Quantification is often subjective, relying on the perspective of trained technicians for identification. This can be particularly important when characterizing weaker blood group interactions, such as the weak subgroup variants of the D antigen. Weaker agglutination of RBCs are visually categorised from 4+ to 1+, while negative RBCs are categorised as 0 (Supplementary Figure 1). This analysis and categorisation is rather arbitrary and completely subjective.

The RhD blood group is the most clinically significant blood group after the ABO blood system, more commonly denoted as ‘+’^1^. While the D antigen usually shows strong haemagglutination in the presence of the corresponding D antibody, there are weaker subgroup variants that do not react as strongly or as readily. These interactions can be difficult to identify using traditional testing since blood group typing is dependent on a simple visual analysis, and can be easily overlooked or misinterpreted^2^. While much more difficult to identify because of their partial antigen or minute interactions, these weaker groups are nonetheless as clinically significant, and can stimulate the formation of antibodies in the recipient which can still result in haemolytic transfusion reactions in subsequent transfusions. This represents a major and unresolved concern in transfusion.

Currently, there are two methods available for quantitative analysis of RBC-IgG antibody interactions and antigen density which are not subjective to human interpretation; 1) flow cytometry, and 2) fluorescence microscopy. Both methods require fluorescence which may affect binding. Flow cytometry measures fluorescent-labelled antibodies attached to blood cells in suspension as the cells pass through a laser in single file^2, 3^. Surface plasmon resonance (SPR) holds advantages over these methods as it measures real time interactions and is label free; it can also be very sensitive.

SPR has been widely utilized for the detection and analysis of interactions between biomolecules^4–17^. SPR can monitor intermolecular binding events in real time, allowing analysis of the interaction kinetics between biomolecules. Whole cell investigations using SPR are significantly less common^12, 18–20^. This is because average cell sizes are orders of magnitude larger (8–15 µm) than the evanescent field depth (~300 nm). Large cells are also unsuitable with the microfluidic system of most commercial SPR instruments as cells can settle or congregate^21^. However, unlike most cells, RBCs are highly deformable in nature to allow for easy vascular transport, which makes its use with microfluidics suitable.

SPR for blood group antigen detection^22, 23^ and antibody detection^24–26^ has been reported. Quinn *et al*.^22^ demonstrated the detection of A and B antigens on whole RBCs using SPR by functionalising the sensor surface using the corresponding blood group IgM antibody^22^. While this method successfully demonstrated selectivity for both A and B antigens, surface regeneration was poor. Harsh regeneration conditions resulted in a loss of antibody/biosensor functionality after a single use. This was due to either the inability to fully desorb bound material or partial removal of the functionalised surface.

More recently, another platform utilizing SPR image analysis demonstrated multiplex RBC typing^23^. This study highlighted the ability to detect the presence of A, M, and D antigens on the RBC surface using each antigen’s corresponding IgG antibody, and provided sedimentation profiles to that of the whole cells flowing through SPR imaging device. However, this study focused on typing the RBCs for antigens rather than quantification, nor did the study mention the detection limits for weaker RBC antigen-antibody interactions.

In our previous study, the concept of using a generic anti-human IgG antibody to detect antigen positive RBCs was explored using the D antigen as an example^27^. The concept draws from the Indirect Antiglobulin Test (IAT) used to detect blood groups using IgG antibodies with the CAT^3^. As anti-human IgG is able to recognize and bind to the Fc region of human IgG antibodies for detection, functionalising the sensor surface with such an antibody allows for the detection of any human IgG antibody. Therefore, when a positive-RBC is sensitised using an IgG antibody and injected over the sensor surface, the anti-human IgG are able to detect the sensitised RBCs, and thereby detect the presence of an antigen. Furthermore, the surface was fully regenerable, showing little to no degradation of the functionalized surface for more than 100 regenerations.

Expression of the D antigen varies greatly from person-to-person, and as SPR is a quantitative technique, a strong correlation with the estimated amount of D antigens expressed and binding response was found. However, sensitivity of this new method for weak and partial antigen detection has not yet been explored^27^. Here, we optimize and validate this new technique using human-sourced donor blood samples to detect the weakest expressions of the D antigen^27^.

There are two main classes of D variants: 1) weak D, and 2) partial D^1^ (Supplementary Figure 2). These variations are mostly caused by mutations within the RHD gene^1^. Weak D class variants still express the entire D antigen, but at estimated low quantities (Table 1). Partial D variants only express D antigens with partially complete structures. As the D antigen is comprised of a mosaic of different epitopes, the absence of one or more of these epitopes often results in weak detection using commercial anti-D IgM^28^.

**Table 1:** Number of estimated D antigen binding sites per red blood cell^29^.

D Antigen	Estimated D sites per cell^29^
Normal D	9,900–33,300
Weak D	66–5,200
Partial D VI	300–14,502

Most partial D antigens are divided into nine categories; however, up to 30 different partial D antigen profiles are characterised, but rarely seen^28^. Category DVI is one of the more prevalent partial D antigen. Both weak D and partially D variants are usually detected using the indirect antiglobulin test (IAT), which is a non-quantitative technique^28^.

Here, we investigate the potential and sensitivity of SPR to detect and quantify weak and partial antigens for blood typing. The first objective of this study is to explore the concept of functionalizing an SPR sensor chip with anti-human IgG to detect RBCs with weak or partial D antigen variants which have been pre-sensitized with anti-D IgG. The second is to determine and optimize the sensitivity of this technique to hopefully provide an attractive alternative that can also quantify the level of interaction. The technique is validated directly with human-sourced donor blood samples – not reagent blood samples. We aim at developing a reliable, sensitive and quantitative analytical method that can be automated for robust blood typing.

## Results and Discussion

**Figure 1:**
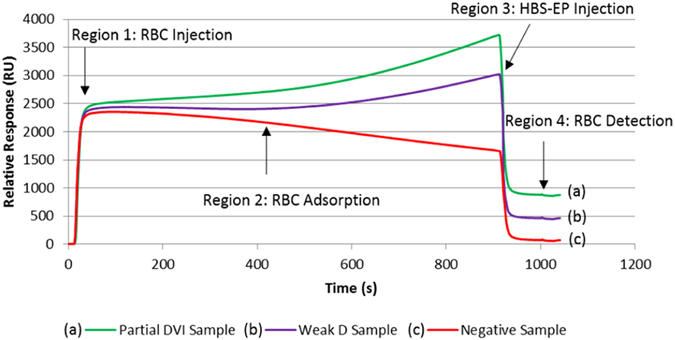
SPR sensogram presenting the injection of RBC over a IgG antibody functionalised sensor surface, and the difference in relative binding response when compared to steady-state running buffer. Depicted here, are (**a**) a partial DVI sample, and (**b**) a weak D sample compared to that of (**c**) a negative sample. Presensitised RBCs were washed 4 times prior to being injected at 10% concentration (v/v) in Celpresol LISS at a rate of 1 µL min^−1^ for 15 min.

A regenerable platform for weaker D variant blood group detection was explored using SPR. A CM5 sensor chip was functionalised with a monolayer of anti-human IgG prior to injection of RBCs that have been sensitised with anti-D sera that is typically used For Further Manufacturing Use (i.e. anti-D FFMU). If positive, the anti-D FFMU selectively binds to the D antigen sites, allowing the Fc regions of the IgG antibodies covering the RBCs to be detected by the generic anti-human IgG immobilised onto the sensor chip surface. Negative RBCs will not bind and are directly eluted. This distinction for weak D and partial D sensogram is shown in Fig. 1. The key question which must be addressed to validate this concept is whether the SPR is sensitive enough to detect and quantify the antibody-antigen interactions of weaker D variants. Each sample was prepared twice, and tested three times with a total of six replicates, during this study to show reproducibility and reliability.

Reagent red cells are commercially standardised for the detection of various clinically significant blood groups, including the D antigen. Each set of reagent red cells comes with two positive D blood samples, with varying expression of D antigen sites, and one negative D sample. The three reagent red cells (Abtectcells) were used as a reference compared to the weaker D variants. In total, 35 human-sourced donor blood samples were tested; 17 weak D variants, 11 partial D category DVI variants, and 6 negative D cells were used as controls.

Each SPR sensogram from Fig. 1 presents four distinct regions of the RBC injection over the sensor surface: 1) a rapid initial rise, corresponding to the introduction of the RBC sample into the SPR detection zone over the functionalized surface. This is caused by the differing refractive index of the RBCs in Celpresol LISS compared to the running buffer, HBS-EP; 2) a pseudo plateau representing the RBC flow over the detection zone at steady state; 3) a rapid decrease region, once the RBC injection ends, illustrating return to the running HBS buffer, and 4) a plateau indicating the final amount of RBC retained over the surface. The difference between the region 4 plateau and the baseline prior to region 1 represents the amount of sensitized RBCs adsorbed upon the functionalised surface, quantified as a binding response (RU). The pseudo plateaux of region 2 varies among samples and represents the reversible adsorption kinetics of RBCs over the surface. Here, the diameter of the analyte probed (RBC, d = 7 μm) is an order of magnitude higher than the thickness (0.3 μm) and two orders or magnitude shorter than the length (L = 1800 μm) of the SPR detection zone. While only 1/20 of the RBC is detectable by SPR, up to 200 cells can be present at any time in the micro-fluidics capillary, therefore producing a good statistical value. However, these unique conditions of macroscopic cell analysis by SPR explain the unsteady rise/decrease observed in region 2, which differ from the traditional plateaux observed with small analytes.

### Detection of Weak D Variants

The weak D phenotype is a result of less antigen sites present on the RBC surface^29^. As with the D phenotype, the weak D antigen consists of all the D epitopes; however, they are expressed weakly. Also similar to the D phenotype, the estimated number of antigen sites on weak D cells is highly variable within the population, congruent with the findings of this study (Table 1).

In traditional blood typing, quantitative analysis for RBC antigen-antibody interaction is observed and rated on a scale from strongest to weakest by 4+ to 1+, respectively (Supplementary Figure 1). This is achieved through basic visual analysis by trained personnel comparing the size of agglutinates formed in the presence of antibodies; the results are empirical. As the D antigen sites upon the RBC can be measured quantitatively, this scale also very roughly indicates the approximate number of potential binding sites.

**Figure 2:**
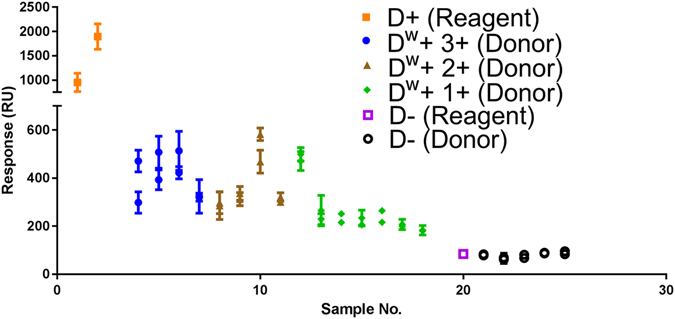
SPR detection of 15 weak D antigens on donor samples using anti-D IgG FFMU compared to 2 D-positive reagent cells, and 6 D-negative samples (1 reagent, 5 donor). Each sample was prepared twice, and each preparation was tested three times; the average and standard deviation are plotted. *Discontinuous Y-axis was used to better show the detection variance between weak D samples. RU measured after t = 900 s when the region 4 plateaux has reached steady-state.

Comparing this scaling system with the results observed from SPR analysis, there was a general trend correlating the strength of binding for each group of samples. RBC incubated with their specific IgG antibody are retained on the anti-human IgG functionalized SPR chip. The SPR detection signal is proportional to the quantity adsorbed and the RU value after the adsorption phase has reached steady-state (region 4) was measured and plotted for each sample in Fig. 2. A large variance between the binding responses for weak D cells was observed with RBC adsorption ranging from 180–580 RU (Fig. 2). This is consistent with the previous study, where ‘normal’ D antigens on donor cells were detected between a range of 530–1200 RU^27^. We propose that responses below 600 RU can be considered weak. Furthermore, all the negative D samples tested showed binding responses below 100 RU, which is also consistent with previous testing. Three reagent cells, two D-positive and one D-negative, were included for comparison. As shown in Fig. 2, the adsorption signal of D-positive cells can be very strong compared to that of weak D, but importantly, SPR analysis is sensitive enough to distinguish between all weak D and negative samples; there is no overlapping of binding response.

Weak D samples categorised with 3+ binding strength showed the strongest binding overall among all the samples. The lowest binding responses were observed for weak D 1+ samples, as categorised by the test tube method, with detection as low as 181 RU. There were two exceptions among the 15 samples that did not conform; one 2+ sample and one 1+ sample.

This variability could be indicative of the weak D genotype as there have been numerous individual types of weak D documented. These are indistinguishable from each other unless the donor’s DNA is genotyped, but the estimated number of antigen sites for each genotype exist with a range of variability^29^. Another more likely possibility could be the very subjective nature of visual categorisation for binding strength using traditional methods. Many factors play into the role when analysing the ‘strength’ of the interactions using the traditional method (Supplementary Figure 1), a method which is conducted by the naked eye. For example, antibody type used (manufacturer and clone), incubation and detection method, reaction time, and individual technique of the analyst. Simple differences such as how rigorously a test tube is shaken during analysis will affect the size of agglutinates observed; too little can appear stronger, too much and analysis can appear weak. Time of contact is another poorly controlled variable. This is where the benefits of detection via SPR microfluidics lies. Two major advantages of SPR analysis for detection and binding strength quantification are the controlled conditions (time, temperature, shear, concentrations) and the completely objective results.

There are three possible explanations for the high variability of certain results. A first – and by far the most likely – is that it reflects the important RBC antigen density variability among the population (Table 1). A second explanation is a possible lack of – or over – sensitivity of the SPR technique. As the SPR is a concentration-dependent technique, any fluctuations in the concentration of a sample could result in an apparent deviance in binding strength. A third explanation for some large variability is due to the SPR microchannel having a limited field of detection from the gold surface (0.3 µm). The comparatively large size of RBCs (6–8 µm), as well as its biconcave shape, could play a role in the amount of adsorbed mass within the detection field, especially given that binding can theoretically occur at any location on the surface of each RBC. The highly deformable nature of the RBCs travelling through the microfluidic channels (L = 1800 μm) of the Biacore X for analysis could result in non-uniform shapes and orientation of the RBC when adsorption occurs. However, it was shown in the previous study that the SPR signal provided by the base of the RBC is directly proportional to the RBC concentration, and therefore the RBC orientation and whether the whole cell is in the detection zone is irrelevant.

SPR detection of weak D class variants is seen to be more sensitive than that of the test tube categorisation. Sample 12 in Fig. 2 provides an example of this increased sensitivity. While the binding strength for sample 12 has been classified as 1+ using the test tube method by a trained-personnel with more than 20 years in the blood banking industry, the SPR has detected a binding strength more comparable with that of category 2+, or even 3+, blood samples. This increased sensitivity could be used to better quantify binding strength of RBC antigen and antibody interactions, while also providing a definitive cut-off between positive and negative samples.

### Detection of Partial D Variants

**Figure 3:**
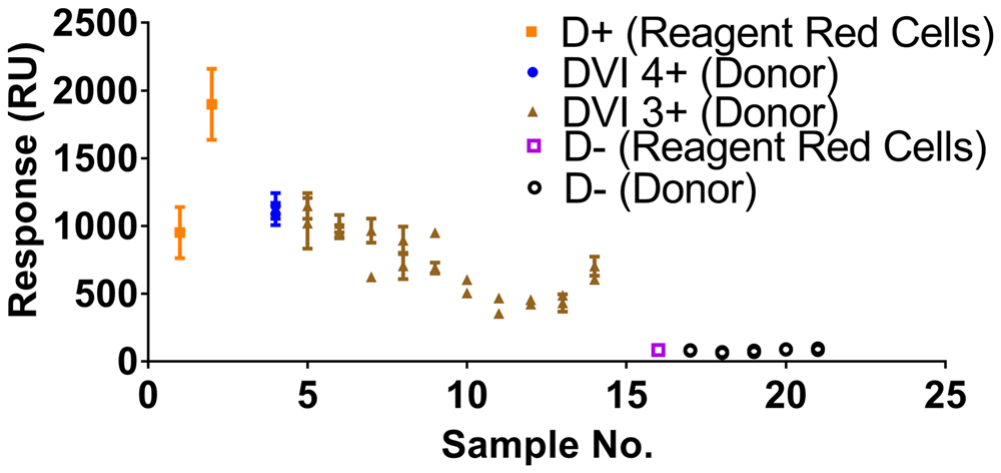
SPR detection of 10 partial D antigens on donor samples using anti-D IgG FFMU compared to 2 D-positive reagent cells, and 6 D-negative samples (1 reagent, 5 donor). Each sample was prepared twice, and each preparation was tested three times; the average and standard deviations are plotted. RU measured after t = 900 s when the region 4 plateaux has reached steady-state.

Partial D variants are D antigens with one or more D epitopes missing from its structure; however, the estimated number of antigen sites is similar to that of cells with the normal D-antigen, and therefore can vary greatly. The partial antigenic structure can lead to extremely weak binding using conventional IgM antibodies, often seen as false negative. However, detection of partial D variants is possible with the indirect antiglobulin test (IAT) using IgG antibodies and anti-human IgG. Among the partial D variants, there are 9 categories, all of which are rare in the human population. However, some are rarer than others. Category D VI (DVI) is one of the more common partial D variants observed. As testing was constrained by the availability of blood samples, 11 donor DVI blood samples were tested in this study (Fig. 3).

There was a clear distinction between positive and negative cells. The general trend of binding response was consistent compared to those cells categorized as 4+ or 3+ using the traditional test tube IAT. 4+ samples showed the strongest binding response, while the responses of 3+ samples were slightly decreased. Also, the responses of 3+ samples of the DVI variants were similar to that of the 3+ sample of weak D variants. The binding responses observed for DVI samples was similar to that of a normal D cell. In fact, the highest DVI sample response was greater than that of the first control for normal D cells, further supporting the correlation between binding response and the number of antigen binding sites.

Similar to previous testing, there was some deviation in binding response among the samples between individual preparations tested for each sample. However, the observed variability between runs for each preparation was noticeably less than for the weak D samples, and even the control samples. As a general observation, cells with higher binding responses showed greater variability than those with lower binding responses. An explanation for this phenomenon could be once again due to the shape of the RBCs. A greater number of binding sites may also increase the possible conformations of RBC adsorption to the sensor surface, accounting for the variability observed.

### Perspectives

SPR analysis as a quantitative blood typing technique has great potential. While current blood typing techniques are both simpler and faster for normal antigenic expression, depending on the patient expression of D, typical anti-D IgM clones used can result in false negative reactions in the presence of weaker D variants; this is particularly true for partial D variants. Detection of weaker blood group variants are more limited and currently still require incubation prior to analysis as they are best detected using polyclonal antibodies, which inherently include IgG. Partial D is only one example of such antigen. Others examples include antigens such as Duffy A and B (Fy^a^, Fy^b^)^28^. Even though SPR analysis is reliant on equipment and expensive consumables, and is confined to a laboratory, the key advantage of this technique is its ability for quantitative detection of blood groups, especially for weaker variants as shown.

Nonetheless, the ability to quantitatively detect both weak D and partial D variants using SPR does hold promise for replacing the current subjective and arbitrary methods of quantifying antibody-antigen interactions for blood groups. While each individual sample cannot truly be compared to each other as expression of antigen density and structure is subject to genetics, general trends can easily be seen. When comparing between, and even among, the weak D and partial D VI samples tested in this study, samples categorised at certain binding strengths do appear to group within certain ranges of binding responses. Though there are exceptions, this overlap could indeed be due to the subjective nature of 4+ to 1+ categorisation.

As weak D cells have a decreased number of binding sites, the range of binding observed was much lower than that of the DVI samples and correlated with categorisation using traditional methods. For an accurate comparison of the apparent binding strength and the number of antigen sites on a cell, a correlation study with the known techniques, such as flow cytometry and fluorescence microscopy, could be explored. The concentration-dependent nature of SPR could produce a standardised curve where the number of antigen sites could be accurately discerned from the binding strength observed.

Although the overlap of the estimated number of D sites per cell between weak D and partial DVI variants is substantial (Table 1) and would negate the ability for distinction between the two, the practiced method for distinguishing phenotype of D variants in a clinical setting is not through detecting the number of D antigens upon the RBCs. Instead, D variants, in particular partial D variants, are detected using specialised antibody kits which contain varying types of D antibodies which show a unique binding pattern for each partial D variant. Using the SPR for specific detection of partial D variants could be further explored through the application of these specialised antibody kits. However, during clinical analysis the type of D antigen, whether it is a normal, weak or partial variant, is not as significant as the actual detection of the presence of a D antigen.

Despite variability between preparations for some samples, especially given the smaller scale of weak D responses, all weak D responses observed were above the 100 RU threshold for D negative samples. This 100 RU threshold provides a good, clear distinction between positive and negative during analysis, particularly when differentiating between weak and negative blood groups. Furthermore, there are indications that SPR detection provides a more sensitive method for quantifying weak interactions for blood groups typing, where positive detection of weak D class variants ranging from 180 RU and above. This is an advancement over the previous study which merely reported detection and optimization of this SPR technique, while also supporting the <100 RU cut-off for negative samples.

There were occasional inconsistencies between sample preparations, or between individual tests from the same preparation; this is likely due to the large size and shape of the RBCs which far extends the limited field of detection from the sensor surface (0.3 µm). Additionally, the sensor surface is only partially covered within the detection field. These critical factors affect whole cell detection and result in decreased sensitivity^22^. A longer microfluidic channel and SPR detection zone could be beneficial.

## Conclusion

We have presented a sensitivity study for blood group detection using surface plasmon resonance (SPR) through functionalization of a gold sensor chip with anti-human IgG. Anti-human IgG can potentially interact with any IgG sensitised RBC by the binding of the Fc region of IgG antibodies. Using amine coupling, anti-human IgG antibody was covalently immobilized to a gold sensor surface. RBCs are pre-sensitized through incubation with a known IgG antibody (i.e. anti-D IgG). Positive pre-sensitised cells will bind to the anti-human IgG monolayer. The change of mass detected and reported as a response unit (RU) (>100 RU). Unbound negative cells are directly eluted (<100 RU). The surface is then regenerated in preparation for a new sample.

The variability measured between different red blood cell samples is substantial. However, strong evidence suggests that this variability simply reflects the wide range of antigen concentration found among the population. Some binding interactions are strong and easily discernible. However, there are weaker variants that can be more difficult to detect. In this study, the binding response and sensitivity of the D variants, weak D and partial D, were explored. Weak D classed cells were detected between a range of 180–580 RU. This is due to the lower expression of antigens upon the surface of the cells. Cells with partial D antigens, category D VI, were also positively identified (352–1147 RU), showing stronger expression similar to that of normal D antigens. Using SPR, the detection of two classes of weaker D variants was achieved with this fully regenerable platform using SPR, adding another level of sensitivity to previous studies. This opens up great new potential for identifying, quantifying and classifying blood group antibody-antigen interactions using controlled conditions and completely objective results. Furthermore, newer models of SPR biosensors can be automated and multiple samples analysed simultaneously, allowing for a quantitative, multi-antigen blood typing platform.

## Materials and Methods

### Chemicals and Equipment

All chemicals and sensor chips were purchased from VWR International (Brisbane, Australia) unless otherwise stated. Anti-D IgG For Further Manufacturing Use (FFMU) antibodies and polyspecific anti-human globulin (AHG) antibodies were supplied by Quotient EU (Edinburgh, United Kingdom). Abtectcell red blood cells (RBCs), Celpresol and Celpresol Low-Ionic Strength Solution (LISS) were supplied by CSL Limited (Melbourne, Australia). Anti-human IgG Fc (Clone CBL101) was purchased from Merck Australia (Melbourne, Australia). EDTA blood samples were sourced from the Australian Red Cross Blood Service (ARCBS) (Melbourne, Australia), stored at 4 °C; samples were analysed within 7 days of collection. The BIAcore X system (GE Healthcare, Uppsala, Sweden) was used for all analyses.

### Methods

Amine coupling was used to immobilize anti-human IgG Fc upon a CM5 SPR chip surface. Each CM5 chip contains a medium molecular weight carboxy methyl dextran layer grafted onto a gold surface. The surface was first activated by injecting an equal volumetric mixture of 100 mM N-hydroxysuccinimide (NHS) and 400 mM 1-Ethyl-3-(3-dimethylaminopropyl)carbodiimide (EDC) at 5 µL min^−1^ for 7 min. Both NHS and EDC solutions were made with distilled water before use, aliquoted and stored at −15 °C until required. Anti-human IgG Fc (0.5 µg mL^−1^) ligand was dissolved in 10 mM sodium acetate buffer and then injected over the functionalised surface for 6 min at 5 µL min^−1^. Unreacted sites were blocked by 1 M ethanolamine-HCl, injecting at 5 µL min^−1^ for 7 min. The achieved binding response of anti-human IgG Fc was approximately 9,700 relative units (RU).

During these experiments, reagent red cells (Abtectcells III 3%) and human-sourced donor red cells were used. Reagent red cells are standardised human red cells which have been washed and kept in a cell stabilisation solution with a constant concentration. Donor whole blood was collected from donors by the ARCB using their standard blood drawing and storage process, including stabilization using Ethylenediaminetetraacetic acid (EDTA). These donor cells were washed using a cell preservation solution, Celpresol, upon receipt for stabilization and to remove plasma, and diluted to 3% solution using Celpresol. These cells have not been commercially standardized.

RBCs were incubated with excess anti-D IgG FFMU for 30 min at 37 °C (1:1 by volume) to sensitize the cells. After sensitization, the cells were washed 4 times using Celpresol LISS and centrifugation before dilution to 10% concentration in Celpresol LISS for injection into the BIAcore X system.

Temperature was kept constant at 37 °C to maximize binding of IgG antibodies which are optimal at this temperature; the human body temperature. A single flow cell detection mode, flow cell 2, was used for binding analysis. Sensograms were analysed with standard BIAcore X control software. Regeneration of the chip surface was completed using 3 M MgCl_2_ at 1 min pulses at 1 µL min^−1^. The general purpose running buffer, HEPES buffered saline (HBS-EP), was used throughout all experiments.

While weaker D variants are comparatively rare, the most commonly seen donor weaker D variants are weak D (D^w+^), and the partial D variant, category DVI. Validation of the quantitative detection of these variants was subject to the availability of donor blood.

Current methods for quantification of binding strength for weaker D variants are completely subjective, relying on the analysis of trained personnel. The SPR results were validated with two traditional quantification methods of RBC-antibody interactions using test tubes. First, each blood sample was incubated with anti-D IgG FFMU at 37 °C for 30 mins. Once sensitized, the RBCs were washed vigorously with PBS solution using a Bio-rad Diacent-CW-12 mm cell washer. Finally, a drop of AHG was added to facilitate agglutination. If positive, clumps (or agglutinates) of RBCs were visible with varying degrees of binding strength and categorised from 4+ to 1+. If negative, no visible binding had occurred and the sample was thus categorised as 0. Each blood sample was prepared twice using exactly the same concentration and buffer solutions, and each preparation was tested three times, therefore providing six replicates, in total. The average and standard deviations are reported. Each sample was tested within 1 week of delivery.

## Electronic supplementary material

Quantitative Detection of Weak D Antigen Variants in Blood Typing Using SPR - Supplementary Material
