# Supplementary material for: Quantitative Detection of Weak D Antigen Variants in Blood Typing using SPR
Source: Sci Rep. 2017 May 9;7:1616. doi: 10.1038/s41598-017-01817-x (PMC5431640; doi:10.1038/s41598-017-01817-x)
Supplement: Supplementary file 1 — Quantitative Detection of Weak D Antigen Variants in Blood Typing Using SPR - Supplementary Material [file 41598_2017_1817_MOESM1_ESM.pdf]

QUANTITATIVE DETECTION OF WEAK D ANTIGEN VARIANTS IN BLOOD TYPING USING SPR

Whui Lyn THEN[1], Marie-Isabel AGUILAR[2] and Gil *GARNIER[1]***

[1]Bioresource Research Institute of Australia (BioPRIA), Australian Pulp and Paper Institute (APPI), Department of Chemical Engineering, Faculty of Engineering, Monash University, Clayton, VIC 3800, Australia.
*gil.garnier@monash.edu +(61) 3 9905-9180
[2]Monash biomedicine Discovery Institute and Department of Biochemistry and Molecular Biology, Faculty of Medicine, Nursing and Heath Sciences, Monash University, Clayton, VIC 3800, Australia,

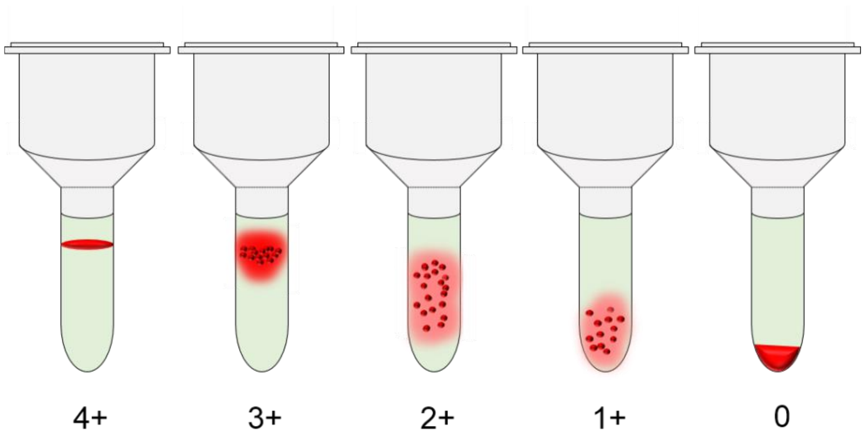

Supplementary Figure 1. Depiction of current technique used for rating blood group antibody-antigen binding strength during a column agglutination test (CAT). Strong clear positive samples are visually categorised as 4+, while weaker positive variants can be categorised between 4+ to 1+, with the latter being the weakest; Negative reactions are denoted as 0.

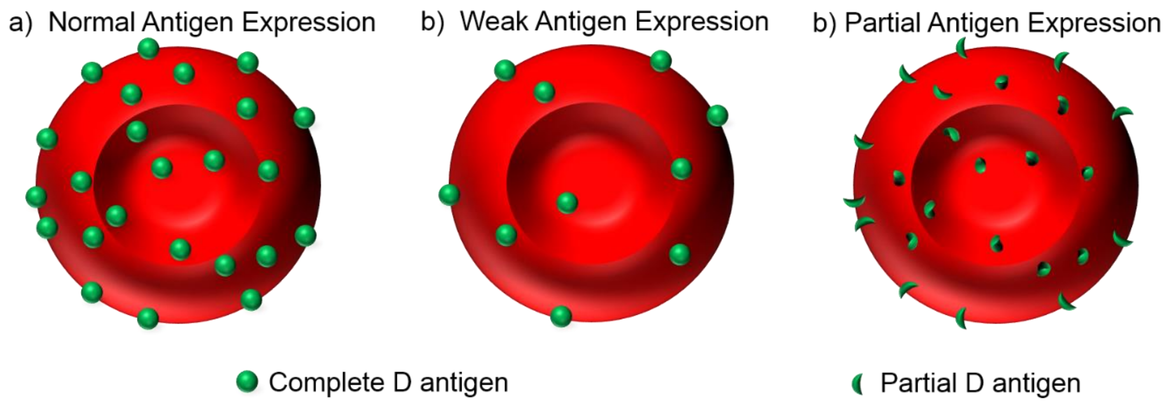

Supplementary Figure 2. Schematic representations of the different types of antigen expression on the surface of red blood cells; a) normal, b) weak, and c) partial expression.
